# *Primula octopoda*, a Stoloniferous New Species From Western Sichuan, China

**DOI:** 10.1002/ece3.74088

**Published:** 2026-08-27

**Authors:** Qin Huang, Xin Wang, Wen‐Jun Huang, Jie‐Yi Yang, Hong‐Li Pan, Jian Ru, Jiang‐Ping Wang, Wen‐Bin Ju

**Affiliations:** ^1^ Ecological Conservation, Restoration and Resource Utilization on Forest and Wetland Key Laboratory of Sichuan Province Sichuan Academy of Forestry Chengdu Sichuan China; ^2^ Sichuan Giant Panda National Park Observation and Research Station Wolong Forest Ecology Observation and Research Station of Sichuan Province Aba Sichuan China; ^3^ Batang County Bureau of Forestry and Grassland Batang Sichuan China; ^4^ Key Laboratory for Regional Plants Conservation and Ecological Restoration of Northeast Jiangxi, College of Life Science Shangrao Normal University Shangrao Jiangxi China; ^5^ Mountain Ecological Restoration and Biodiversity Conservationey Key Laboratory of Sichuan Province, Chengdu Institute of Biology Chinese Academy of Sciences Chengdu Sichuan China

**Keywords:** molecular phylogeny, morphology, new species, *Primula*, taxonomy

## Abstract

*Primula octopoda* W.B.Ju & Q.Huang, a new species of *Primula* (Primulaceae) in sect. *Aleuritia*, is herein described and illustrated from Batang County, Sichuan Province, southwestern China. Morphologically, *P. octopoda* is most similar to *P. stenocalyx*, but it can be readily distinguished by its dwarf habit, the presence of stolons 2–7 cm in length ending in small rosettes occasionally bearing a single flower, elliptic to broadly obovate leaf blades, a very short or absent scape bearing one or two flowers, and a smaller, annulate corolla with a prominent yellow eye. The complete chloroplast genome of *P. octopoda* spans 154,164 bp with a GC content of 36.97% and contains 125 genes. Phylogenetic analyses using plastid and ITS data both place *P. octopoda* within sect. *Aleuritia* while supporting its distinct species status. Given the current lack of adequate information on its population size and geographic range, the species is provisionally assigned a status of Data Deficient (DD) according to the IUCN Red List criteria.

## Graphical abstract

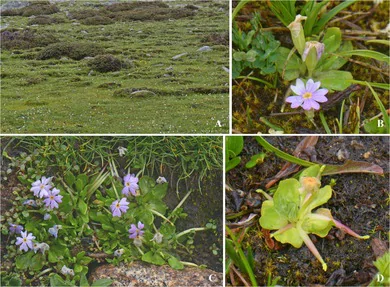

## 1 Introduction

*Primula* Linnaeus (1753: 142), the largest and most widespread genus in Primulaceae, comprises more than 500 species worldwide (Richards 2003). The genus is primarily distributed across the temperate and alpine zones of the Northern Hemisphere, with a few species occurring in Ethiopia and Southeast Asia, and a single isolated species found in South America (Hu 1990, 1994; Hu and Kelso 1996; APG IV 2016). Its greatest species diversity is concentrated in the Sino‐Himalayan region, particularly in southwestern China, while a limited number of taxa extend to mountainous areas of tropical Asia, South America, and East Africa (Richards 2003). China harbors more than 300 *Primula* species, with the highest diversity found in the southwestern provinces, especially Yunnan, Sichuan, and Tibet (Hu and Kelso 1996; Richards 2003). According to the Flora of China, sect. *Aleuritia* comprises 49 species in China (Hu and Kelso 1996). Among these, Sichuan Province plays a crucial role as a center of *Primula* diversification (Yang, Zhang, et al. 2023). Western Sichuan lies within the Hengduan Mountains, a global biodiversity hotspot that supports remarkable levels of plant endemism and ecological heterogeneity (Hu 1994; Hu and Kelso 1996). In recent decades, intensive botanical exploration in this region has led to the discovery and description of numerous new *Primula* taxa (Xu et al. 2014, 2016, 2017, 2019; Xu, Hu, and Hao 2015; Xu, Liu, et al. 2015; Ju et al. 2018, 2021, 2023; Yuan et al. 2018; Li et al. 2023; Luo et al. 2023).

In June 2025, an unusual population of *Primula* was discovered during a field expedition in Batang County, Sichuan Province. Morphological observations revealed glabrous plants with leaf rosettes lacking basal scales, abaxial leaf surfaces densely covered with white farina, bracts typically swollen at the base to form a sac‐like structure, a tubular calyx, a funnelform corolla with lobes deeply emarginate or bifid at the apex, and an oblong capsule slightly exceeding the calyx in length. These combined characters confirm its placement in *Primula* sect. *Aleuritia* Duby (1844: 41). After consulting relevant literature (Smith and Fletcher 1944; Hu 1986, 1990; Fang 1994, 2003; Hu and Kelso 1996; Wu 1999; Richards 2003) and examining herbarium specimens (BM, CDBI, E, GH, K, KUN, NY, P, PE, US, and WU), we concluded that this taxon is morphologically distinct and represents a previously undescribed species based on morphological and phylogenetic evidence. It is most similar to *P. stenocalyx* Maximowicz and is therefore described here as a new species.

## 2 Materials and Methods

### 2.1 Taxonomy and Morphology

The morphological description and illustration of the new species were based on observations of fresh specimens collected during field investigations and on the examination of herbarium material, including type specimens deposited at CDBI. Morphological characters were described using the terminology adopted in the *Flora of China* (Hu and Kelso 1996). The classification of *Primula* adopted in this study follows Hu and Kelso (1996) and Richards (2003). For comparative purposes, digital images of herbarium specimens particularly type specimens were consulted through several online databases, including the Chinese Virtual Herbarium (http://www.cvh.ac.cn/), JSTOR Global Plants (https://plants.jstor.org/), the Global Biodiversity Information Facility (https://www.gbif.org/), and Europeana (https://www.europeana.eu), with a focus on collections from BM, CDBI, E, GH, K, KUN, NY, P, PE, US, and WU. In total, approximately ca. 70–90 herbarium specimens, including type specimens and identified collections of morphologically similar species, were examined. The conservation status of the new species was assessed following the criteria and categories of the IUCN Red List (IUCN Standards and Petitions Committee 2024).

### 2.2 Sequencing, Assembly, and Annotation

Total genomic DNA (gDNA) was isolated from dried *Primula octopoda* leaves following an adjusted CTAB protocol (Porebski et al. 1997). The extracted gDNA was then fragmented enzymatically into pieces ranging from 200 to 500 bp. Subsequent steps included end repair, addition of a poly‐A tail, and ligation of sequencing adapters. A small‐fragment DNA library was prepared through PCR amplification and sequenced on an Illumina NovaSeq 6000 instrument (Illumina, San Diego, CA, USA) using paired‐end 150 bp mode. To ensure data quality, reads containing adapters, with more than 20% of bases showing a Phred score below 5, or with over 10% ambiguous bases (N) were filtered out using fastp v0.23.2 (Chen et al. 2018). The chloroplast (cp) genome and the Internal Transcribed Spacer (ITS) regions were assembled using the *embplant_pt* and *embplant_nr* databases in GetOrganelle v1.7.7.7.1 (Jin et al. 2020). Annotation of the cp genome was performed with PGA (Qu et al. 2019), using *Primula farinosa* as a reference. Pseudogenes on the cp genome were identified using Geneious Prime 8 software (https://www.geneious.com). Finally, a circular visualization of the complete chloroplast genome was generated with OGDRAW v1.3.1 (Greiner et al. 2019).

### 2.3 Phylogenetic Relationship Reconstruction

To conduct phylogenetic analysis, besides the chloroplast genome and ITS sequences of *Primula octopoda*, we extracted the ITS sequence of *P. stenocalyx* and the cp genomes of *P. kialensis* and *P. membranifolia* , all belonging to section *Aleuritia*. We also retrieved from GenBank (https://www.ncbi.nlm.nih.gov/genbank/) the ITS sequences of 70 *Primula* species (representing 4 subgenera and 28 sections) and the chloroplast genomes of 32 species (spanning 4 subgenera and 18 sections; Table S1). All sequences were aligned using MAFFT and subsequently refined with Gblocks v0.91b (Castresana 2000), Maximum likelihood (ML) trees were inferred with IQ‐TREE v2.1.4 (Nguyen et al. 2015), using *Androsace integra* and *A. paxiana* as outgroups (Table S1). Final tree visualization and layout were performed in FigTree v1.4.4 (Rambaut 2018; available at https://github.com/rambaut/figtree/releases/tag/v1.4.4).

## 3 Results and Discussion

### 3.1 Characteristics of the Cp Genome

**FIGURE 1:**
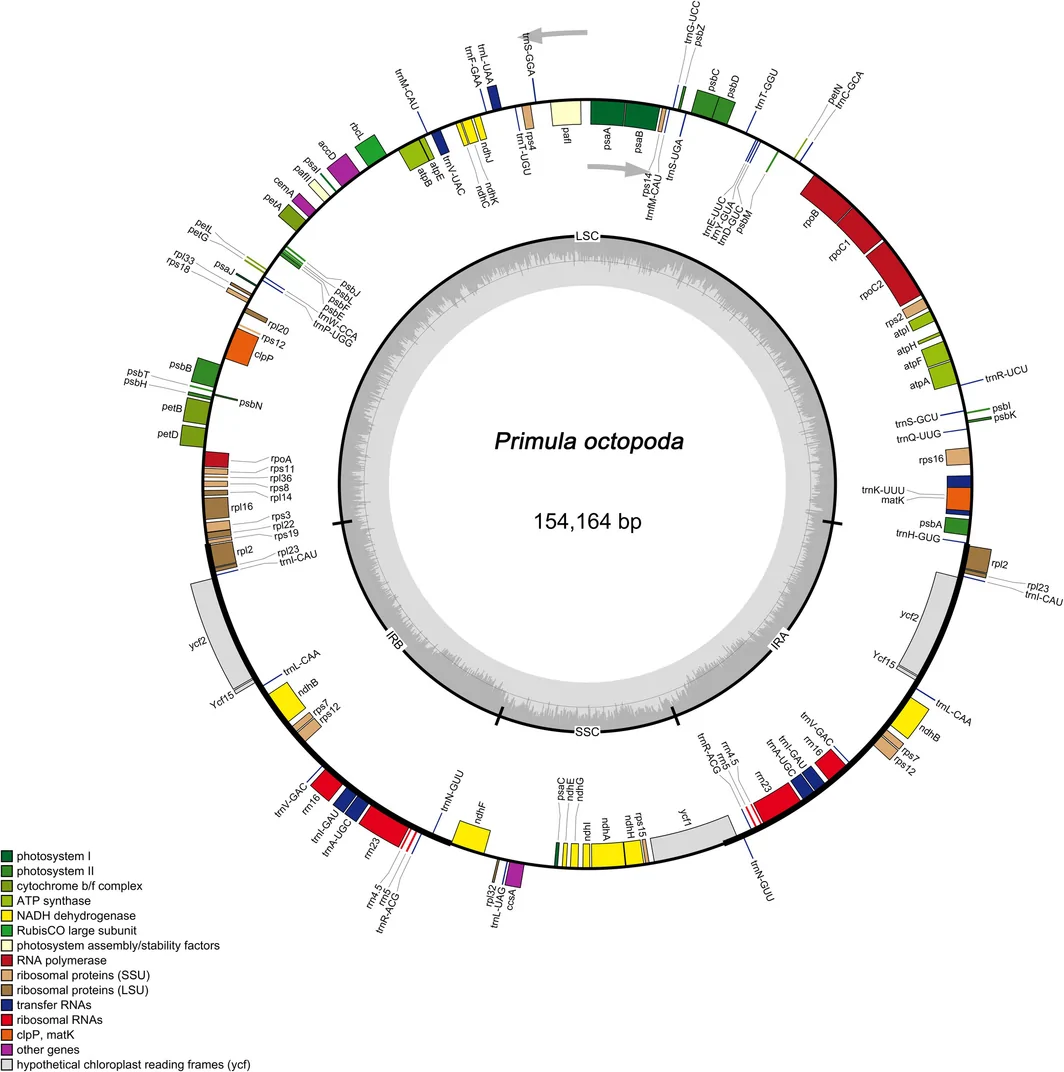
The complete chloroplast genome map of *Primula octopoda*. The outer circle shows distribution of genes (different colors represent different roles). The arrows indicate the genes' transcription directions inside and outside the circle, respectively. The inner circle shows a typical quadripartite structure with a large single‐copy (LSC) region, a small single‐copy (SSC) region, and a pair of inverted repeats (IRA and IRB) regions.

The chloroplast genome of *Primula octopoda* was 154,164 bp in length and displayed the conventional quadripartite structure comprising two inverted repeats (IRa and IRb, each 25,826 bp), a large single‐copy (LSC, 84,728 bp), and a small single‐copy (SSC, 17,784 bp) region (Figure 1). This quadripartite organization, consisting of two inverted repeats separated by large and small single‐copy regions, is a typical feature of most angiosperm plastomes (Ruhlman and Jansen 2014), and has also been documented in other *Primula* species, such as *P. sinensis* (Liu et al. 2016), *P. obconica* (Zhang et al. 2019), and *P. medogensis* (Li et al. 2024). The overall guanine‐cytosine (GC) content was calculated to be 36.97%. This content varied across regions, measuring 34.83% in the LSC, 30.31% in the SSC, and 42.76% within the IRs. Genome annotation identified 129 genes, encompassing 85 protein‐coding sequences, 36 tRNAs, and 8 rRNAs (Figure 1; Table S2).

### 3.2 Phylogenetic Relationship

**FIGURE 2:**
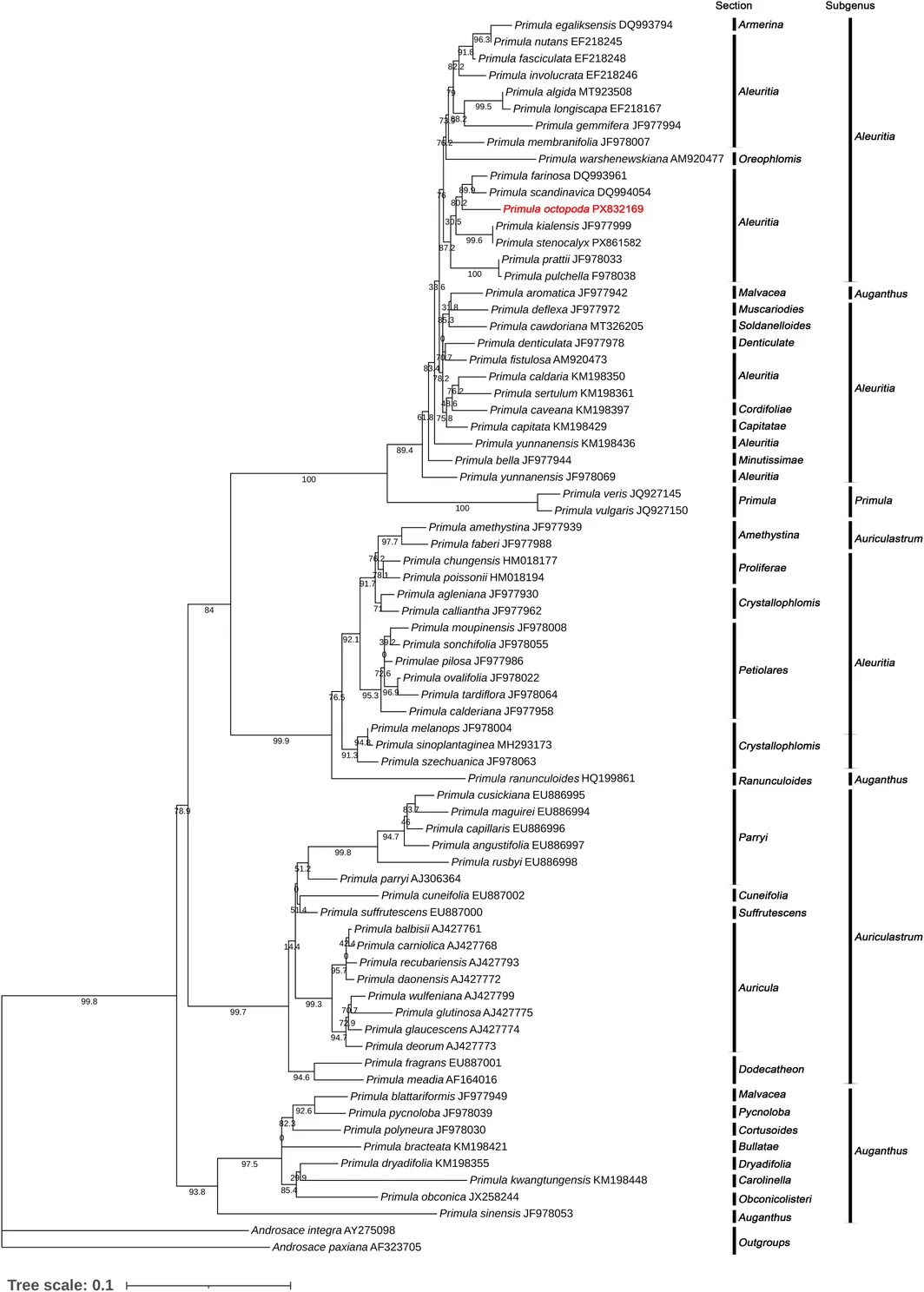
Phylogenetic relationships of 72 *Primula* species inferred from Internal Transcribed Spacer sequences. Numbers at nodes indicate bootstrap values and the scale bar (0.1) represents the number of substitutions per site.

**FIGURE 3:**
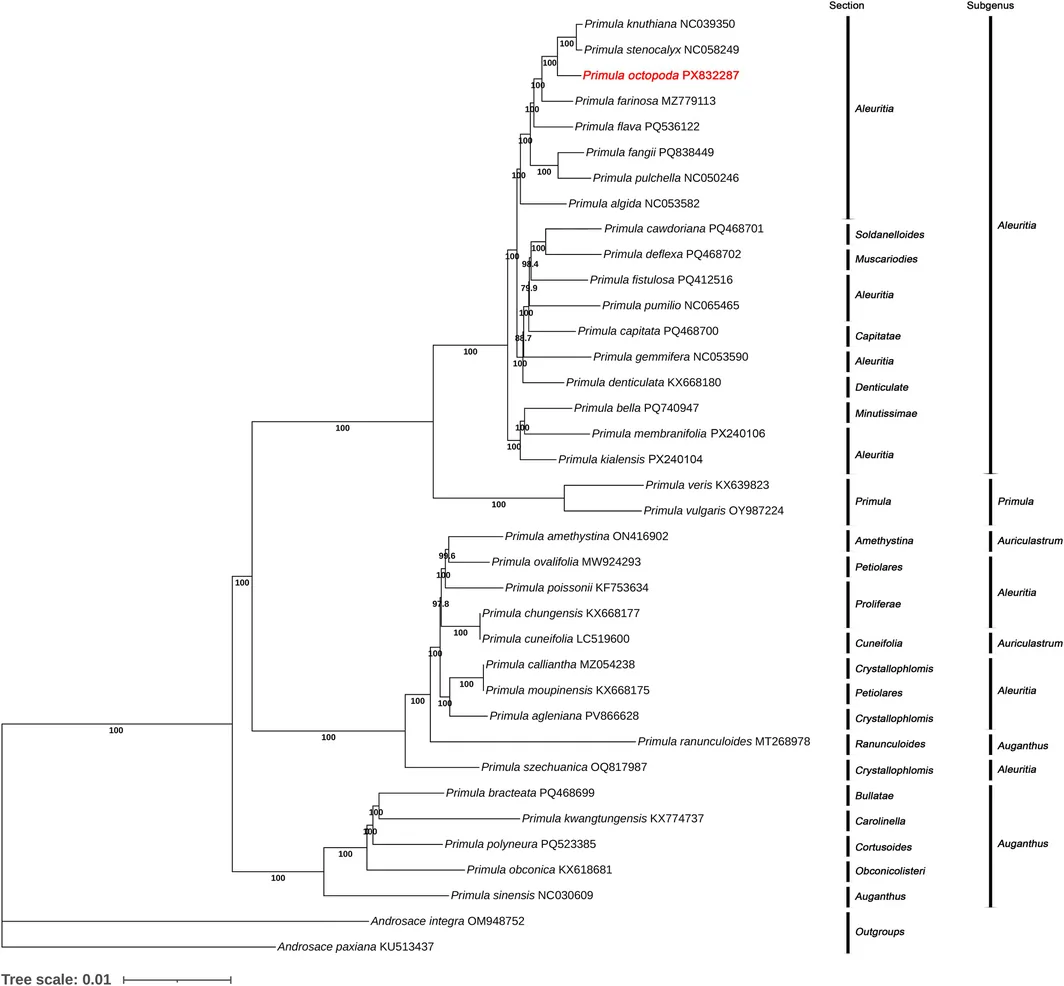
Phylogenetic relationships of 35 *Primula* species inferred from complete chloroplast genome sequences. Numbers at nodes indicate bootstrap values and the scale bar (0.1) represents the number of substitutions per site.

Based on phylogenetic analyses using both chloroplast genome and nuclear ITS sequence data, the systematic position of the newly described species *Primula octopoda* was clarified. In the ITS phylogeny (Figure 2), *P. octopoda* is resolved within a clade containing *P. farinosa* and *P. scandinavica*. This group, in turn, forms a sister clade to another subclade comprising *P. stenocalyx* and *P. kialensis*, all belonging to section *Aleuritia*. In the chloroplast phylogeny (Figure 3), *P. octopoda* is placed within a larger clade of section *Aleuritia* species, including *P. knuthiana*, *P. stenocalyx*, *P. farinosa* , *P. flava* , *P. fangii*, *P. pulchella* , and *P. algida* . Within this clade, while *P. octopoda* shows a closer association with *P. stenocalyx* and *P. knuthiana* compared to other species, it does not form a strictly resolved sister‐species relationship with any single taxon, instead occupying a distinct branch. This pattern supports its genetic distinctiveness at the plastid level. Our phylogenetic analyses, based on both ITS and chloroplast genome data, largely corroborate the framework of previous phylogenetic studies on *Primula*, in which major sections and subsections of the genus are generally well resolved (Conti et al. 2000; Mast et al. 2001).

Morphological similarity does not necessarily imply a close phylogenetic relationship, as convergent or parallel evolution may cause distantly related species to evolve similar morphological traits (Li 2022). Notably, the phylogenetic relationships inferred from the biparentally inherited nuclear ITS marker are not fully congruent with the strong morphological similarities observed between *P. octopoda* and *P. stenocalyx*. The chloroplast topology suggests a closer affinity to *P. stenocalyx* than the ITS tree indicates. This discordance may reflect complex evolutionary histories such as incomplete lineage sorting, hybridization, or chloroplast introgression. Cytonuclear discordance has been widely documented in plants (e.g., Rieseberg and Soltis 1991; Maddison 1997), including within *Primula* (Mast et al. 2001). Despite these inter‐marker inconsistencies, *P. octopoda* maintains a unique and distinct phylogenetic position in both analyses, unequivocally corroborating its status as a distinct evolutionary lineage and a separate species.

## 4 Taxonomic Treatment

**FIGURE 4:**
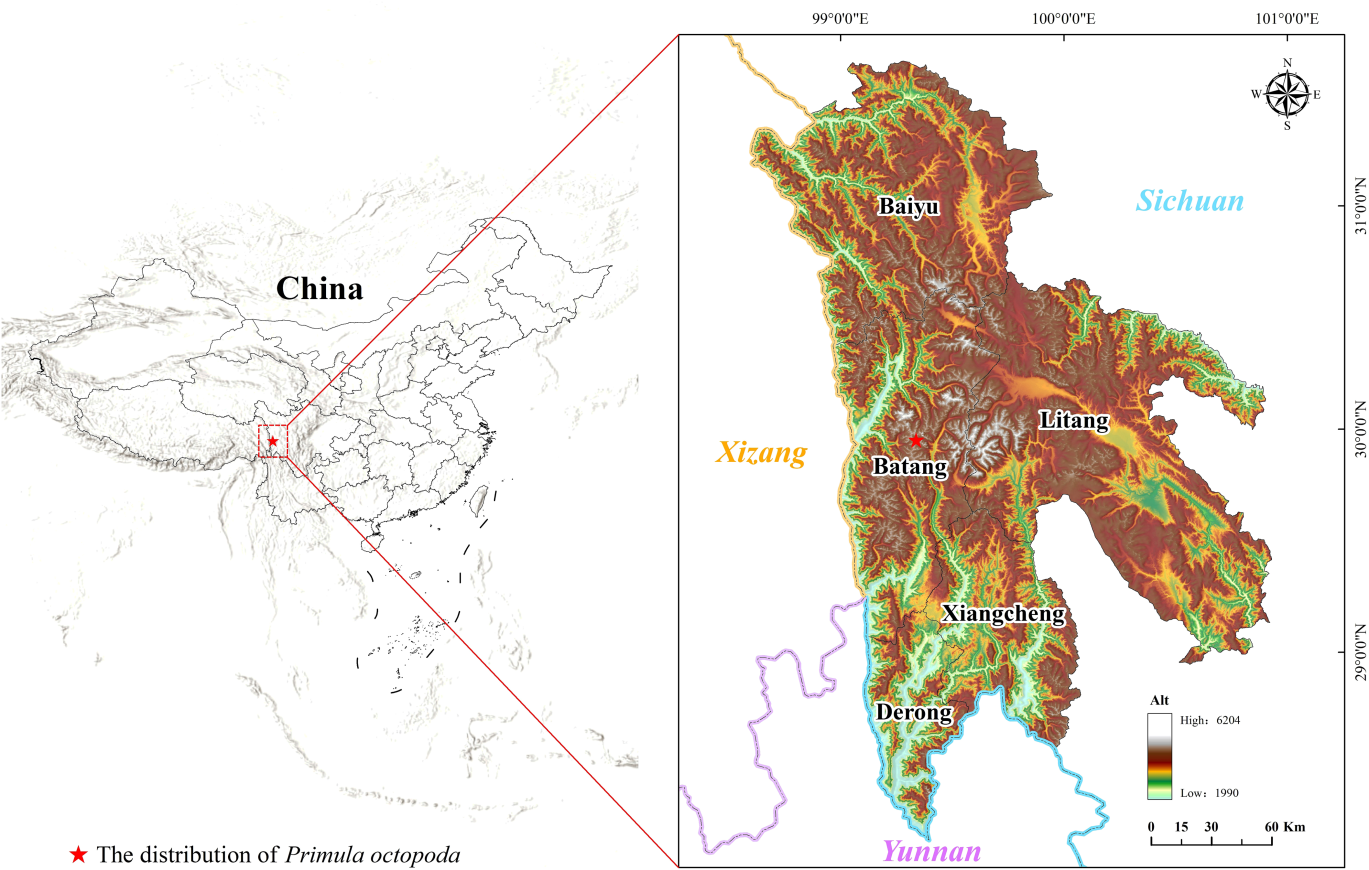
The distribution of *Primula octopoda* W.B.Ju & Q.Huang, in Batang County, Sichuan Province, China.

**FIGURE 5:**
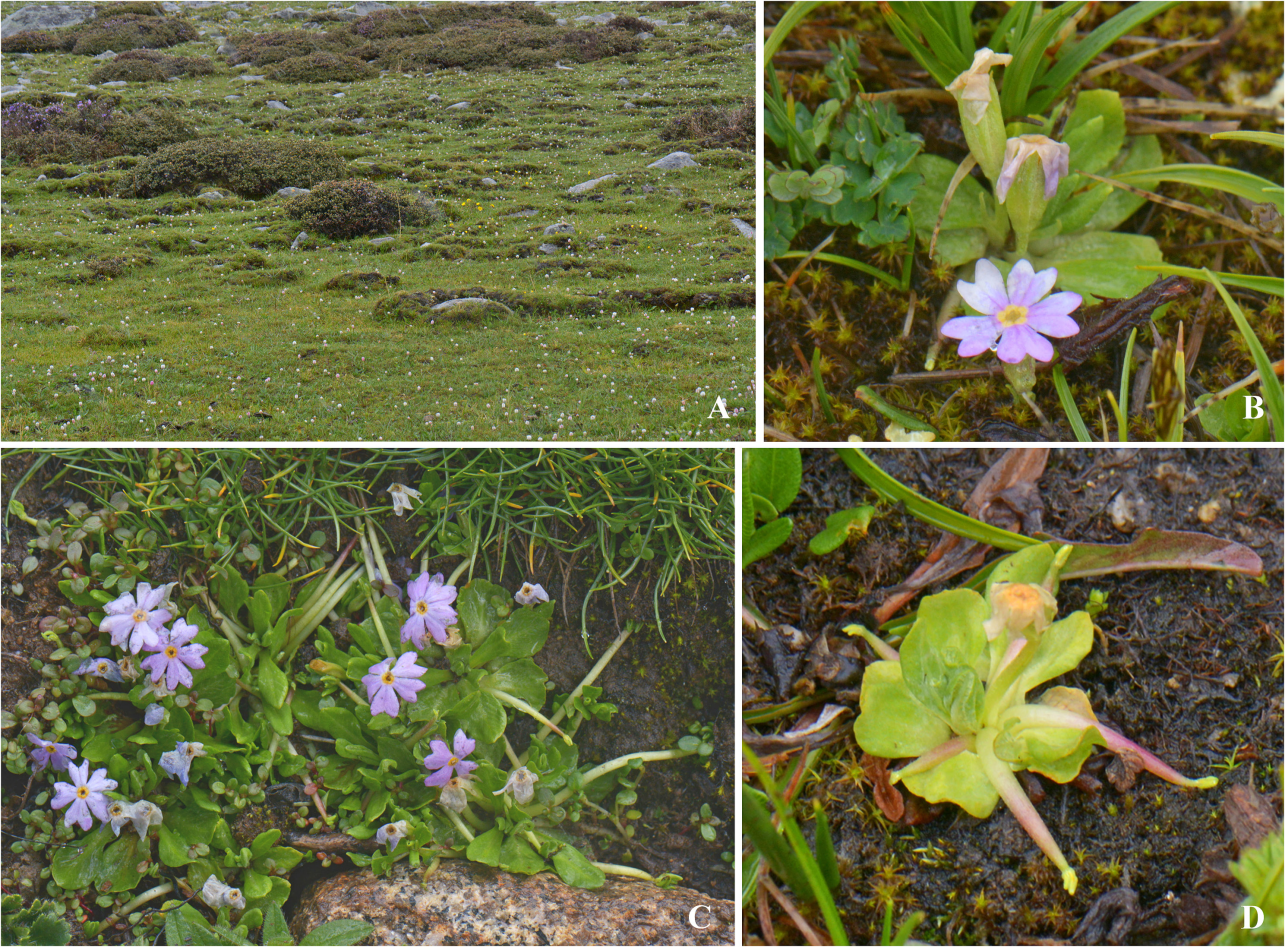
Habitat of the *Primula octopoda*. (A) Habitat; (B–D) Plants.

**FIGURE 6:**
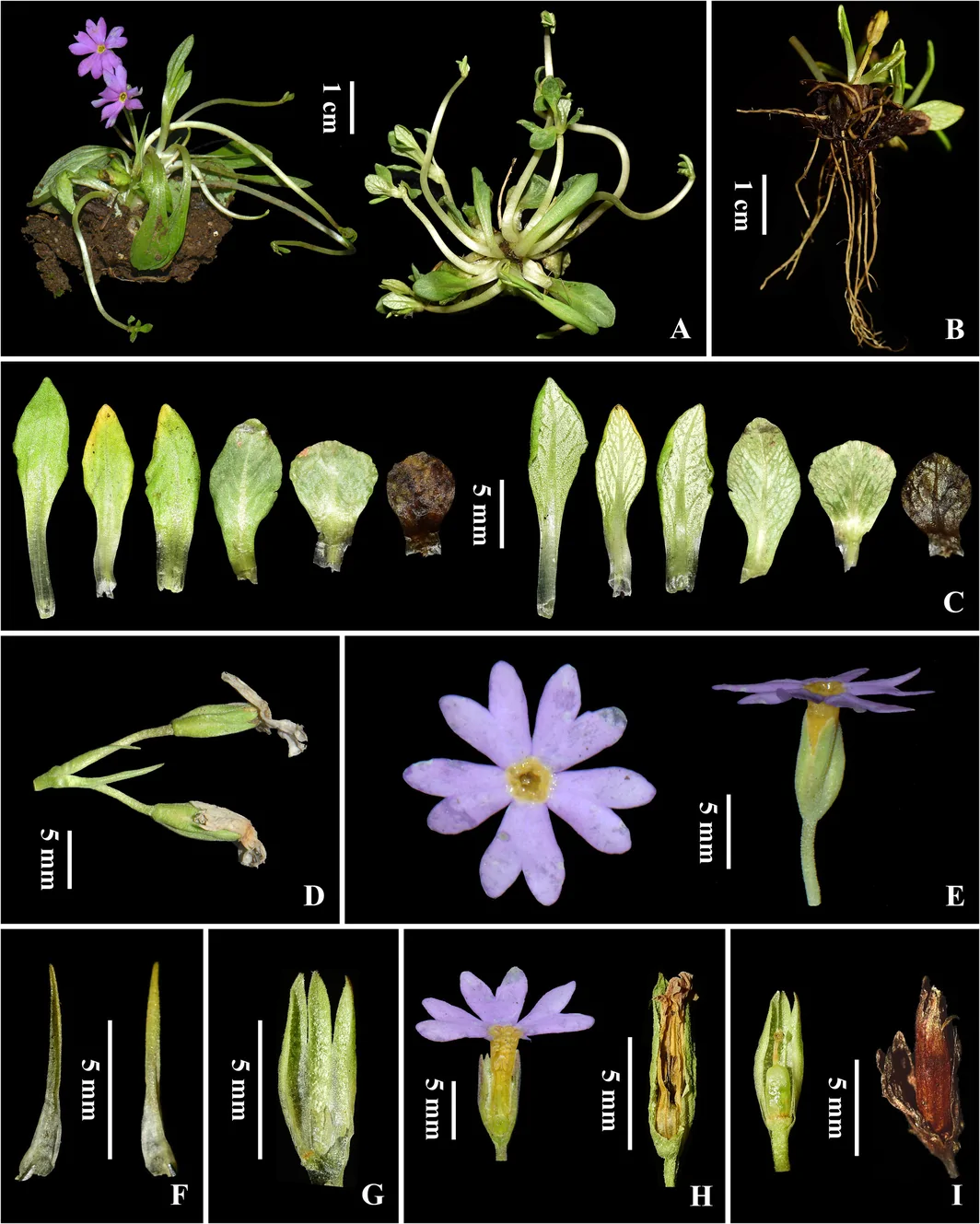
Morphology of *P. octopoda*. (A) Plant; (B) roots; (C) leaves; (D) inflorescence; (E) corolla; (F) bracts; (G) calyx; (H) thrum flower and pin flower; (I) capsule.

**FIGURE 7:**
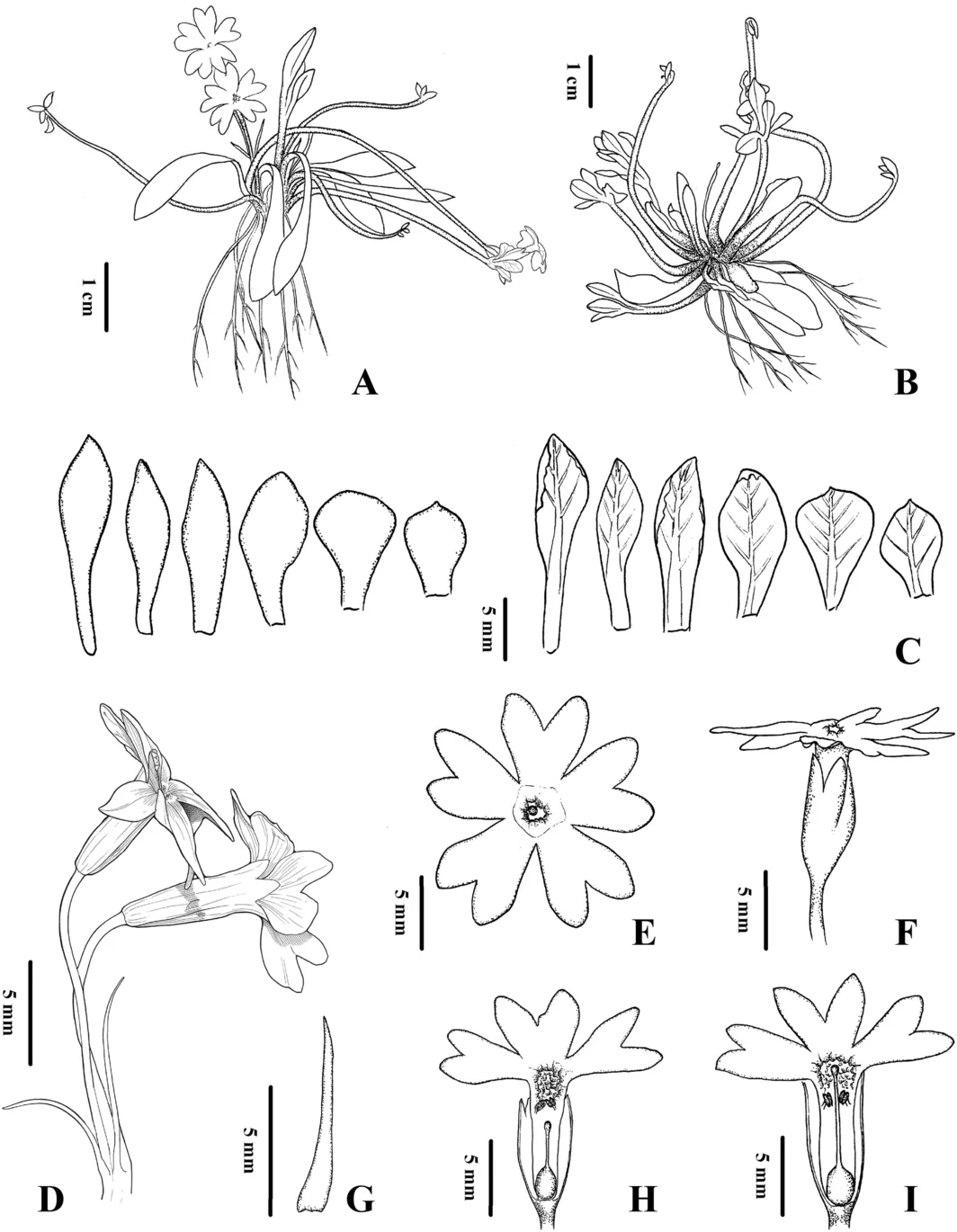
Morphology of *P. octopoda*. (A, B) Plant; (C) leaves; (D) inflorescence; (E, F) corolla; (G) bracts; (H) thrum flower; (I) pin flower. Drawn by Mr. Zhen‐Long Liang based on the holotype.

**FIGURE 8:**
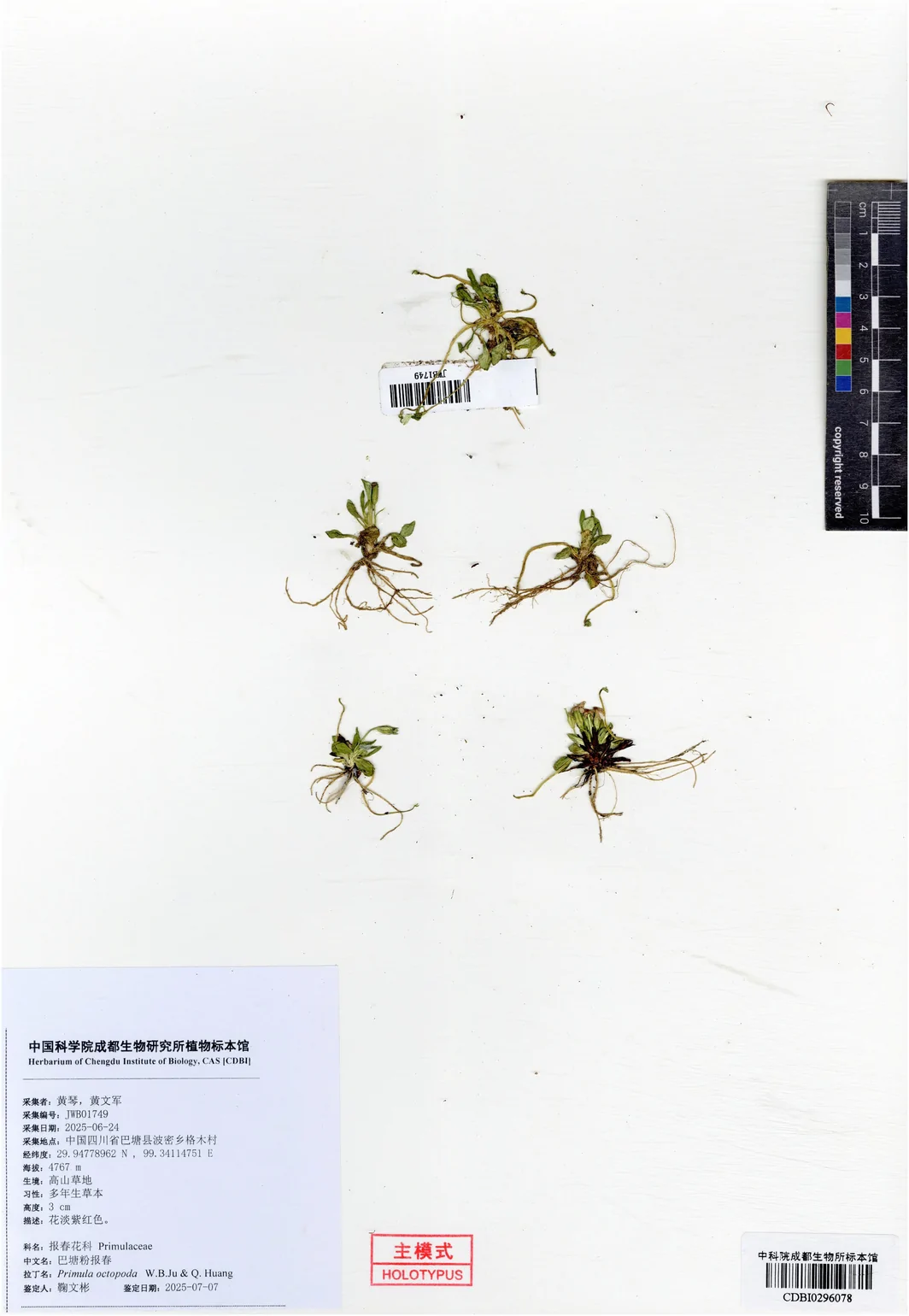
Holotype image of *P. octopoda*.

**Primula octopoda W.B.Ju & Q.Huang, sp. nov**. Figures 4, 5, 6, 7, 8

**Taxonomic and Morphological Notes**. In this study, the newly described species *Primula octopoda* produces short stolons (2–7 cm in length), each terminating in a small rosette occasionally bearing a single flower, exhibiting a typical clonal reproductive trait. This strategy likely facilitates rapid local spread and population renewal. Thus, stolons are not only a key morphological trait in certain *Primula* lineages but also an essential component of their ecological adaptation and reproductive strategy (Smith and Forrest 1923; Ju et al. 2021; Wu et al. 2023; Watt 1882; Klimešová and Klimeš 2007). Morphologically, *P. octopoda* is most similar to *P. stenocalyx* Maximowicz 1881, sharing narrowly lanceolate bracts, a tubular calyx with similarly divided oblong or lanceolate lobes, stamens and style included within the corolla tube, and an oblong capsule, yet it differs by its dwarf stature, reduced or absent scape, and production of stolons terminating in small rosettes. A detailed morphological comparison among these two species is given in Table 1.

**TABLE 1:** The comparison of morphological characters of *Primula octopoda* and *P. stenocalyx*.

Characters	*Primula octopoda*	*P. stenocalyx*
Stolons	2–7 cm long, each terminating in a small rosette and occasionally bearing a solitary flower	None
Leaf blade	Elliptic or broadly obovate, 1.0–2.0 × 0.5–1.2 cm	Obovate to oblanceolate or spatulate, 1–5 × 0.5–1.5 cm
	Margin entire	Margins entire, or crenulate to dentate
	Abaxially white farinose	Abaxially efarinose or white or yellow farinose
Scape	Up to 2 cm long or absent, 1–2 flowered	1–15 cm long, 2–8 (10) flowered
Corolla	Pinkish purple with a yellow eye	Bluish lavender to pink with a white eye
	Annulate	None
	Tube 0.7–0.9 cm, limb 1.0–1.6 cm wide	Tube 0.9–1.5 cm, limb 1.5–2.0 cm wide

In the genus *Primula*, the formation of stolons plays a biologically and ecologically significant role. Stolons are specialized vegetative structures that extend horizontally from the parent plant and give rise to new individuals at their nodes. This clonal growth strategy greatly enhances a plant's ability to expand within its local habitat and maintain population stability. In particular, stolons facilitate spatial spread and improve survival in challenging environments such as alpine meadows, rock crevices, or moist limestone cliffs covered with mosses, especially where sexual reproduction via seeds may be unreliable or limited (Klimešová and Klimeš 2007). Currently, two modes of stolon formation have been observed in *Primula* species. The first involves stolons directly emerging from the leaf rosette and spreading horizontally around the plant. Examples include *Primula caldaria* W.W. Smith & Forrest (1923: 35), *P. dujiangyanensis* W.B. Ju, Bo Xu & X.F. Gao (2021: 275), and *P. luquanensis* Z.K. Wu & Wei Zhou bis (2023: 534) from section *Aleuritia* Duby, as well as *P. tenella* King ex G. Watt (1882: 13) of section *Minutissimae* Pax (1889: 202). The second mode occurs when the fruiting scape comes into contact with the soil, where roots and new leaf rosettes develop at the bract insertion points, forming new plantlets. This has been documented in *P. bracteosa* Craib (1917: 250) of section *Petiolares* Pax (1889: 173), and *P. weimingii* Bin Yang & Y.H. Tan (2023: 435) of section *Obconicolisteri* Balfour (1913: 141). Species utilizing either strategy often form dense clonal colonies under shady, moist forest valleys or on wet rocky slopes, enhancing their adaptation to harsh habitats.

The stoloniferous trait of the newly described *Primula octopoda* provides a key new case for understanding the diversity of clonal reproductive strategies and their adaptive significance within the genus. This study finds that the stolons of *P. octopoda*, which arise directly from the basal leaf rosette and terminate in new daughter rosettes, clearly align with the first mode described above. However, compared to other known stoloniferous species in the genus, *P. octopoda* exhibits a suite of extreme morphological adaptations: a highly dwarfed stature, a drastically reduced or absent scape resulting in nearly sessile flowers, and notably short stolons (only 2–7 cm). This combination of traits represents a highly specialized adaptation to its native high‐altitude scree or rock crevice habitats. In such harsh environments characterized by strong winds, low temperatures, and unstable, nutrient‐poor substrates, the diminutive growth form and clonal strategy effectively reduce mechanical stress, exploit localized microsites, and ensure population persistence and local expansion where seed reproduction may be limited.

Overall, the morphological and phylogenetic data presented here support the recognition of *P. octopoda* as a distinct species, and its stoloniferous habit represents an adaptive strategy to the harsh alpine environments of the Hengduan Mountains.

**Diagnosis**. The new species most closely resembles *P. stenocalyx*, sharing narrowly lanceolate bracts, a tubular calyx with similarly divided oblong or lanceolate lobes, stamens and style included within the corolla tube, and an oblong capsule. However, it differs in its shorter stature, the presence of 2–7 cm long stolons each ending in a small rosette and occasionally bearing a solitary flower, elliptic to broadly obovate leaves, a scape up to 2 cm long or absent with 1–2 flowers, and an annulate corolla with a yellow eye and smaller flowers (Figures 2 and 3).

**Type**. CHINA. Sichuan: Batang county, Bomi township, Gemu village, 29°56′42.58″ N, 99°20′32.36″ E, 4767 m alt., 24 June 2025 (fl.), *Qin Huang & Wenjun Huang JWB01479* (***holotype***: CDBI!; ***isotype***: SCFI!).

**Description**. Perennial herb, dwarf and tufted, to 1.5 cm tall, with flowering plants reaching 3.0–4.0 cm in height. ***Stolons*** leafless, stout, fleshy, flagellate, 2–7 cm long, arising from the base, each terminating in a small rosette and occasionally bearing a solitary flower. ***Rhizome*** very short, base covered with withered remnants of old leaves, without basal scales. Roots few, fibrous, glabrous. ***Leaves*** forming a basal rosette; leaf blade elliptic or broadly obovate, 1.0–2.0 × 0.5–1.2 cm, base cuneate, margin entire, apex obtuse to subrounded, gradually attenuate narrowly winged petiole 0.4–1.5 cm long, green, narrowly winged; glabrous; abaxially densely white farinose, adaxially efarinose; midvein prominent abaxially; lateral veins 4–5 pairs. ***Scapes*** 0–2 cm long, 1–2 per plant, arising from the center of the leaf rosette, shorter than the leaves, sparsely covered with minute glandular trichomes, each bearing 1–2 flowers. ***Bracts*** 2, narrowly lanceolate, 5–15 mm long, slightly swollen at base, glabrous. ***Pedicels*** 0.6–2.5 cm long, sparsely covered with minute glandular trichomes, not elongating in fruit. ***Calyx*** tubular, 6–9 mm long, sparsely glandular trichomes outside, parted to 1/3 to 1/2 of its length, 5‐ribbed; lobes oblong or lanceolate, apex acute to obtuse. ***Corolla*** pinkish purple with a yellow eye, annulate, limb 10–16 mm across, funnelform; lobes spreading, obovate, 4.5–7.5 × 3.5–6.5 mm, deeply emarginate. ***Flower*** heterostylous, Pin flowers: corolla tube 7–9 mm long, widely ampliated above the insertion of stamens; stamens ca. 3 mm above base of corolla tube; style ca. 2/3 as long as tube; Thrum flowers: corolla tube 7–8 mm long, widely ampliated above the insertion of stamens; stamens inserted ca. 2/3 as long as tube; style ca. 3 mm. ***Capsule*** oblong, ca. 5–7 × 3–4 mm, pale brown when mature, glabrous, equaling or slightly exceeding the calyx at maturity.

**Distribution and Habitat**. The new species is currently known only from the type locality, Gemu Village, Bomi Township, Batang County, Sichuan, China (Figures 1 and 2), where it inhabits alpine meadows at elevations of 4700–4800 m. The discovery of *Primula octopoda* adds to the growing number of *Primula* species known from the Hengduan Mountains, underscoring the region's significance as a center of diversification and endemism within the genus. This area, particularly western Sichuan, exhibits pronounced environmental heterogeneity, supporting the persistence and radiation of narrowly distributed alpine taxa (Hu and Kelso 1996).

**Phenology**. Flowering occurs in June; fruiting is unknown.

**Etymology**. The specific epithet of the new species refers to its distinctive feature of producing multiple stout, fleshy stolons that resemble the tentacles of an octopus; hence the name octopoda, which vividly reflects the plant's numerous stolons extending like “eight legs.”

**Vernacular Name**. Chinese mandarin: “bā táng fĕn bào chūn” (巴塘粉报春), meaning “Batang Farinose Primrose” in English.

**Provisional Conservation Status**. Data Deficient (DD). Due to limited field surveys, current knowledge regarding the natural distribution and population size of this species remains inadequate. Consequently, there is insufficient information to reliably evaluate its risk of extinction, either directly or indirectly. In accordance with the International Union for Conservation of Nature Red List Categories and Criteria (IUCN 2024), this species is provisionally classified as Data Deficient. Future comprehensive field investigations, particularly in the high‐altitude regions of western Sichuan and the adjacent Tibet area, are necessary to better understand its population dynamics and geographical range.

## Author Contributions

**Qin Huang:** investigation (equal), writing – original draft (lead), writing – review and editing (lead). **Xin Wang:** visualization (equal), writing – review and editing (supporting). **Wen‐Jun Huang:** investigation (supporting), methodology (supporting), writing – original draft (supporting). **Jie‐Yi Yang:** investigation (equal). **Hong‐Li Pan:** investigation (equal). **Jian Ru:** visualization (supporting). **Jiang‐Ping Wang:** project administration (lead). **Wen‐Bin Ju:** writing – original draft (supporting), writing – review and editing (supporting).

## Funding

This study was supported by the Biodiversity Survey Project of Batang County, Sichuan Province (CLYGZ‐202429), the 2024 Special Program for Precision Collection of Plant. Specimen Resources, National Plant Specimen Resource Center (NPSRC) (E0117G1001), and by the National Key Research and Development Program of China (grant no. 2024YFF130670103).

## Disclosure

Use of AI: No use of AI was reported.

## Ethics Statement

The authors have nothing to report.

## Conflicts of Interest

The authors declare no conflicts of interest.

## Supporting information


**Table S1:** The species used for phylogenetic tree construction and the corresponding accession numbers of ITS and chloroplast genomes.
**Table S2:** Summary of gene contents present in the Primula octopoda chloroplast genome.

## Data Availability

The Internal Transcribed Spacer sequences of *Primula octopoda* and *P. stenocalyx*, along with the chloroplast genomes of *P. octopoda*, *P. kialensis* and *P. membranifolia* have been deposited into the National Center for Biotechnology Information (NCBI) database. Their accession numbers are PX832169, PX861582, PX832287, PX240104, and PX240106, respectively.
